# The Anesthetic Strategy for Patients with Mucopolysaccharidoses: A Retrospective Cohort Study

**DOI:** 10.3390/jpm12081343

**Published:** 2022-08-21

**Authors:** Hsuan-Chih Lao, Ying-Chun Lin, Muh-Lii Liang, Ying-Wei Yang, Ya-Hsien Huang, Ying-Lun Chan, Yung-Wei Hsu, Shuan-Pei Lin, Chih-Kuang Chuang, Jen-Kun Cheng, Hsiang-Yu Lin

**Affiliations:** 1 Department of Anesthesiology, MacKay Memorial Hospital, Taipei 10449, Taiwan; 2 Institute of Brain Science, College of Medicine, National Yang Ming Chiao Tung University, Taipei 11203, Taiwan; 3 Department of Medicine, MacKay Medical College, New Taipei City 25245, Taiwan; 4 Department of Neurosurgery, Mackay Memorial Hospital, Taipei City 104217, Taiwan; 5 Department of Pediatrics, MacKay Memorial Hospital, Taipei 10449, Taiwan; 6 Department of Medical Research, MacKay Memorial Hospital, Taipei 10449, Taiwan; 7 The Rare Disease Center, MacKay Memorial Hospital, Taipei 10449, Taiwan; 8 Department of Infant and Child Care, National Taipei University of Nursing and Health Sciences, Taipei 11219, Taiwan; 9 College of Medicine, Fu-Jen Catholic University, Taipei 24205, Taiwan; 10 MacKay Junior College of Medicine, Nursing and Management, Taipei 11260, Taiwan; 11 Department of Medical Research, China Medical University Hospital, China Medical University, Taichung 40402, Taiwan

**Keywords:** mucopolysaccharidoses, anesthesia, anesthetic risk, airway management, endotracheal tube, respiratory adverse event, cardiovascular adverse event, neuraxial anesthesia

## Abstract

Anesthesia for patients with mucopolysaccharidoses (MPS) is quite challenging due to vital systemic dysfunction following progressive accumulation of lysosomal glycosaminoglycans. Previous studies focused on perioperative difficult airway management under general anesthesia but rarely depicted the concern of choosing the size of the endotracheal tube (ETT) as well as neuraxial anesthesia. This study aimed to analyze the overall anesthetic management and related complications for a thorough anesthetic strategy. Within the study period from 2002 to 2021, each record of the anesthetic and perioperative quality assurance/improvement system for patients with a diagnosis of MPS at MacKay Memorial Hospital was retrospectively reviewed. A total of 51 individuals with 151 anesthesia for 163 interventions were cohort studied, and there were 136 general anesthesia and 15 neuraxial anesthesia. We found that the most common interventions for MPS patients were otolaryngological surgeries (49.6%). Additionally, a secured airway played a marked preference for the most general anesthesia (87.1%). The incidence of difficult intubation was 12.5%. In view of ETT size, a smaller than estimated size was used in MPS type II, III, IV, and VI patients and also in patients who received intubation with multiple attempts. However, a larger than estimated size of ETT was adopted whilst choosing cuffed ones. For neuraxial anesthesia, two failed spinal anesthesia procedures were converted to general anesthesia and 73 percent of the patients received perioperative sedation. In conclusion, through the individualized anesthetic strategy and build-up of an experienced team for airway management, high-quality anesthesia can be ensured in each patient.

## 1. Introduction

Mucopolysaccharidoses (MPS) are a group of inherited lysosomal storage disorders, caused by deficiencies of the enzyme involved in the degrading of glycosaminoglycans (GAGs) [1] and recognized as seven types and 11 subtypes. The incidence of MPS in Taiwan from the Nationwide Newborn Screening Program is varied between each type, which is 0.67 for MPS I, 2.92 for MPS II, and 4.13 for MPS IVA per 100,000 live births, respectively [2]. After the implementation of Taiwanese National Health Insurance in 1995, the number of diagnoses for MPS increased to 8–17 patients every year, contrary to less than three patients diagnosed per year prior to 1995 [3]. Since the initiation of enzyme replacement therapy in 2002 at MacKay Memorial Hospital in Taiwan, the clinical medical service, including the anesthetic management for many interventions in addition to various surgeries, has grown. Anesthesia for patients with mucopolysaccharidoses is quite challenging due to various systemic dysfunctions following progressive accumulation of lysosomal GAGs. There are special considerations for anesthetic management according to different systemic involvement of each types of MPS; of these, airway management would meet the highest risk of mortality and morbidity [4]. The most awkward airway management may occur in type I, II, IV, and VI patients which is similar to the most frequent otorhinolaryngological manifestations [5].

Our previous case series for ten MPS children who received surgical interventions between 1998 and 2000 showed that simultaneous difficult mask ventilation and tracheal intubation occurred in one out of ten patients, leading to devastating mortality [6]. In recent decades, devices for airway management have been greatly improving, such as the invention of the supraglottic airway and video-assisted laryngoscopy, so more flexible anesthetic plans could be devised. In addition to general anesthesia with great concerns of airway management, neuraxial anesthesia for MPS patients may be preferable for surgeries of the lower body. However, there were only few case reports [7,8,9,10,11,12,13] of successful regional anesthesia performed contrast to few cases of failed attempts [14,15] and one case of catastrophic sequala [16]. This study aimed to exploit and analyze anesthetic management and related complications from our experience in the past 20 years for a thorough anesthetic strategy for patients with MPS.

## 2. Patients and Methods

### 2.1. Ethics Statement

This retrospective, single-center study was approved by the Ethical Committee of the Medical Board of MacKay Memorial Hospital, Taiwan (22MMHIS154e).

### 2.2. Data Acquisition

The electronic medical records were systematically reviewed for patients with a diagnosis of MPS within the study period from 2002 January to 2021 December. From all the medical records of the MPS patients, each individual registry in the electronic anesthetic records and quality assurance/improvement (QA) system from the Anesthesiology Department was included in this study. Therefore, the records were only analyzed when the licensed anesthesiologists performed the anesthesia. Anesthesia for interventions by the pediatricians or other physicians would be excluded. Baseline characteristics of the patients, procedural and anesthetic related data, and peri-procedural outcomes were collected.

### 2.3. Anesthesia Method

General anesthesia and neuraxial anesthesia were reported and analyzed separately. For general anesthesia, implementation of airway management included bag-mask ventilation (BMV), tracheal intubation, supraglottic airway (SGA), and pre-existing airway. Spontaneous breathing combined with intermittent BMV was classified as BMV. Tracheal intubation was facilitated by direct laryngoscopy, video-assisted laryngoscopy, flexible fiberscope, or rigid fiberscope. Cases that did not necessitate intubating a secured airway (e.g., neuraxial anesthesia, bag-mask ventilation, and pre-existing airway) were not included in the multivariate analysis. For neuraxial anesthesia, the dose of local anesthetics, perioperative sedation or analgesia, and incidence of conversion to general anesthesia were collected.

### 2.4. Outcome Measures

Data collection for outcome measures included periprocedural respiratory adverse events, cardiovascular adverse events for each anesthetic intervention, and airway management difficulties for general anesthesia.

Difficulties of airway management: documented Cormack–Lehane classification (C/L) III° or IV° [17], events for difficult airway managing including difficult bag-mask ventilation, ≥3 attempts with the primary airway technique, documented prolonged intubating procedure over 30 min, and high air leakage clearly documented over a long time period during ventilation and conversion to a different airway technique.

Respiratory and cardiovascular adverse events: Respiratory adverse effect included documented hypoxia (any occurrence of low partial pressure of oxygen in the arterial blood less than 60 mmHg or an arterial oxygen saturation of less than 90% over 3 min), hypercapnia (end-tidal carbon dioxide pressure > 60 mmHg for over 10 min or any above 75 mmHg), aspiration, reintubation, severe laryngospasm, and postextubation stridor. Cardiovascular adverse events included documented hypotension (decrease 20% of systolic blood pressure (BP) from the baseline or adult mean BP less than 65 mmHg over 10 min), new episode of myocardial ischemia/infarction, resuscitation, requirement of multiple or high doses of inotropic drugs (e.g., atropine, epinephrine, or norepinephrine) for bradycardia or hypotension not directly related to a surgical complication.

### 2.5. Specification of Endotracheal Tube

For the group of patients that received tracheal intubation, the exact inner diameter (ID) of the endotracheal tube (ETT) used would be compared with the estimated size, which was as follows: (1) 3.5 mm for newborns and infants at the age until 6 months old; (2) 4.0 mm for infants older than 6 months; (3) size calculated by Cole’s formula [18] for children older than 1 year: ID in mm = 0.25 × (age in years) + 4; (4) 6.5 mm for female adolescence and adults; and (5) 7.0 mm for male adolescence and adults. The ETTs with or without inflatable cuffs were also analyzed. There were two indicators for observing the chosen size of ETT. First, discrepancy of size was defined as the difference between the exact and estimated size, which was smaller or larger than 0.5 mm of ID. Secondly, difference between the exact and estimated size of the endotracheal tube would be calculated and as a category variant. For each cuffed endotracheal tube, adding 0.5 mm for calculating the difference of size would be manipulated: Difference of size (mm) = documented final size (+0.5 for cuffed tube) −estimated size.

### 2.6. Statistical Analysis

Continuous data are presented as means with ranges. Categorical data are presented as exact numbers of incidents. RStudio Desktop 2022.07.0+548 (RStudio, Boston, MA, USA) was used to perform the statistical analysis for multiple variable linear regression. Statistical significance was set at *p* < 0.05. Prism 8.0.1 (GraphPad Software, San Diego, CA, USA) was used to graph the data.

## 3. Results

### 3.1. Anesthesia Methods and Intervention Procedures

Medical records of 137 patients with MPS diagnosis were reviewed between the study period. Within this group, there were 51 individuals who underwent 151 anesthesia procedures. Twelve general anesthesia were performed for the combined two different surgical or diagnostic interventions. Therefore, there was a total of 163 interventions. The distribution of anesthesia methods for the different types of intervention procedures is summarized in Table 1. Two trials of a supraglottic device which were not listed were converted into endotracheal intubation: one failed due to a severe air-leak and another one used intubating LMA after multiple attempts. Except for diagnostic magnetic resonance imaging (MRI), a secured airway, which was endotracheal intubation, tracheostomy, or pre-existing endotracheal tube, was preferred as the most general anesthesia (87.1%).

Most neuraxial anesthesia procedures were for inguinal hernioplasty (12/15: 80%); the others were for umbilical hernioplasty, incision of hymen, and heel cord lengthening. Combined with low dose sevoflurane or sedation were common and are discussed in Section 3.3. Two failed episodes of spinal anesthesia were converted into general anesthesia with SGA and were not counted as the category of neuraxial anesthesia.

**Figure 1:**
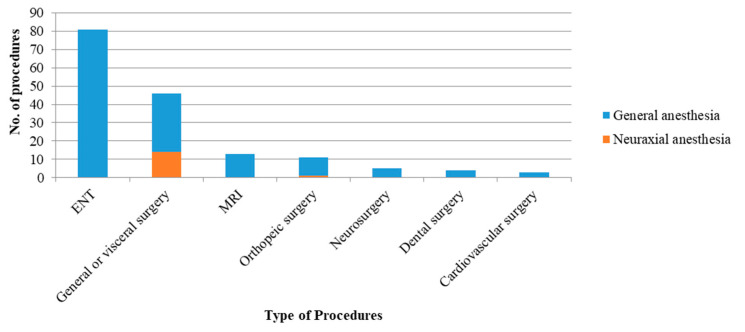
Number of various intervention procedures under general (blue bar) or neuraxial (orange bar) anesthesia.

From the surgical or procedural point of view (Figure 1), the most common interventions for MPS patients were surgeries of ear, nose, and throat (49.6%), which included ventilation tube insertion or removal, adenoidectomy, tonsillectomy, supraglottoplasty, laryngoplasty, tracheostomy, tracheal dilatation or stent placement, and ventilation bronchoscopy. In addition, the general or visceral surgeries, including inguinal hernioplasty, port-a-cath insertion or removal, umbilical hernioplasty, gastrostomy, jejunostomy, and laparoscopy for gastric volvulus, were the second most frequent interventions (28.2%) for these patients. Excluding MRI examinations (7.9%), orthopedic surgeries, including trauma-related surgeries, total knee arthroplasty, surgeries for scoliosis, ankle fusion, and femoral epiphyseal stapling were the third most common surgeries (6.7%). Otherwise, neurosurgeries, dental surgeries, and cardiac surgeries accounted for less than five interventions during these two decades.

### 3.2. General Anesthesia

#### 3.2.1. Distribution of Subtypes of MPS Patients Underwent General Anesthesia

According to the anesthetic records within the past 20 years, general anesthesia for MPS patients with type IVA (25.7%), type II (24.2%), and type I (19.1%) would be the most common in our clinical practice (Table 2). General anesthesia for female patients was only predominant in type I patients, whereas male patients were predominant in other types of MPS, even excluding MPS type II, which is an X-linked disorder. Notably, one female type I patient received eight surgical procedures during her childhood, and another female adult patient received five surgeries. The ages of these patients were all younger than 40 years old (y/o), and there were 38 general anesthesia procedures (27.9%) which were performed on patients younger than 6 y/o, 39 (27.9%) for patients between 6–12 y/o, and 59 (43.4%) for adolescent and adult patients. Moreover, the demographic data including gender, age, body weight, body height, and body mass index (BMI) by the grouping of MPS subtypes is listed in Table 2. Additionally, the information of body height was only available in 94 records, so the valid data number was specified. Comparing data with type I and II patients, older age distribution, shorter stature, and higher BMI in type IVA patients was noted.

#### 3.2.2. Airway Management

Total intravenous anesthesia under intermittent BMV procedures were performed most often in one MPS type IIIB patients (Table 2; 60%). BMV was adopted predominantly for MRI examination (Table 1; 80%). Though frequent nasal or oral suctions for secretion might prolong the procedural duration, no adverse events were noted in those records.

In spite of one failed episode of airway management with SGA, a supraglottic device offered a quick airway for two failed neuraxial blocks. SGA was more frequently applied (40%) in general anesthesia for MPS type II patients. Of these, one episode of hypercapnia was noted in a patient who received umbilical hernioplasty.

There were 19.1% patients who had tracheostomies or previous ETT intubations before the intervention procedures. Besides, endotracheal intubation played the most important role in securing an airway in general anesthesia (58.8%). Intubation was facilitated mostly by direct laryngoscopy (42.5%), then by flexible or rigid bronchoscopy (35%), and least by the video-assisted system (22.5%).

#### 3.2.3. Difficulties of Airway Management

Cormack–Lehane classifications were documented in a total of 60 anesthetic records, and nearly half of them (46.6%) were classified as C/L III° or IV. Most upper airways of C/L III° or IV were MPS type I, whereas some were MPS type II, IVA, and VI. 

Between the 12 anesthesia procedures where occurrence of difficult airway management occurred, there were 10 episodes of ≥3 attempts with the primary airway technique, one episode of conversion from SGA to ET tubes, and the other one, an episode of difficult bag-mask ventilation. Therefore, the incidence of difficult intubation was 12.5%. Furthermore, in the 10 records of multiple intubating attempts, eight of them were classified as C/L III° or IV, whereas two were initially classified as C/L II° prior to intubation. Of note, only one event met the criteria of hypoxia. 

#### 3.2.4. Respiratory and Cardiovascular Adverse Events

Two hypercarbia episodes occurred in MPS type II and IVA patients, and one episode of hypoxia due to difficult mask ventilation in a 12 y/o MPS type II patient. 

Cardiovascular adverse events occurred in five general anesthesia procedures and distributed in MPS type I, II, IIIB, and IVA patients. The most devastating event was sudden cardiac arrest at about one hour after anesthetic induction in a 27 y/o MPS type I patient, who was scheduled to receive aortic valve replacement for critical aortic stenosis and passed away within 72 h after the surgery. Other events included one episode of bradycardia, two episodes of tachycardia, and one episode of hypotension, all of which were reversible during the operation.

Postoperative intensive care was common (39.7%), mostly because of scheduled delayed extubation for airway mucosa decongestion after ENT surgeries (39 in 54). Other reasons for ICU admission were mainly for prolonged spine surgery, neurosurgeries, and open heart surgeries.

#### 3.2.5. Specification of Endotracheal Tube

**Figure 2:**
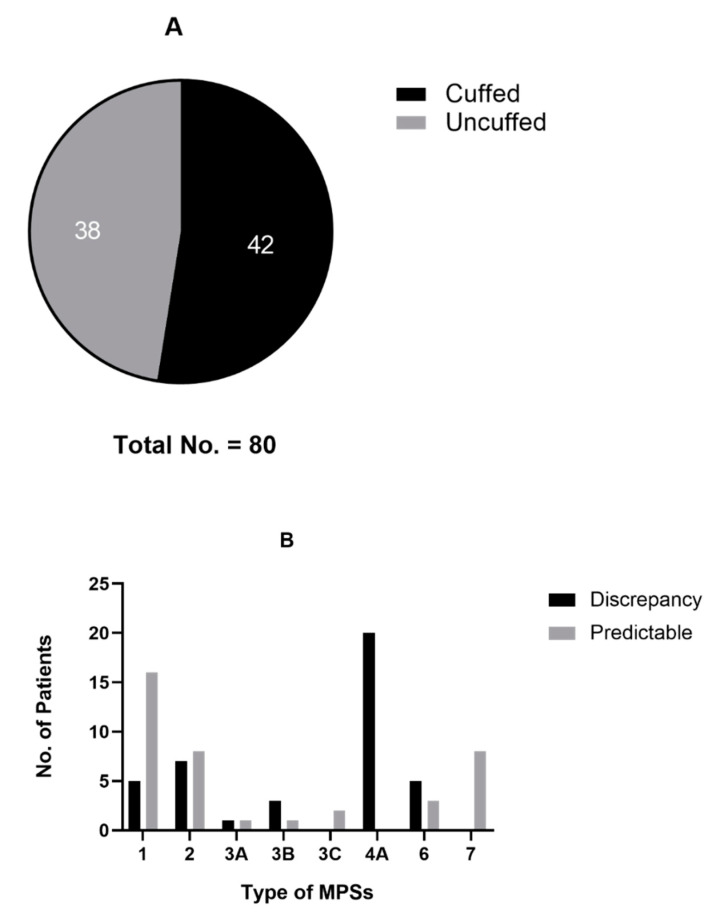
Choice of endotracheal tube: (**A**) Number of cuffed or uncuffed tubes. Cuffed endotracheal tube (black) was lightly preferable (52.5%) to uncuffed (grey) tube; (**B**) Discrepancy of tube size. Inner diameter smaller or larger than 0.5 mm of estimated tube size was recognized as discrepancy (black bar).

A total of 80 general anesthesia events were performed with endotracheal intubation. Cuffed endotracheal tubes were used in more than half of them (Figure 2A). By means of comparing the actual size of the endotracheal tube to estimation, a discrepancy was especially noted in patients with MPS type IV A (Figure 2B). 

**Figure 3:**
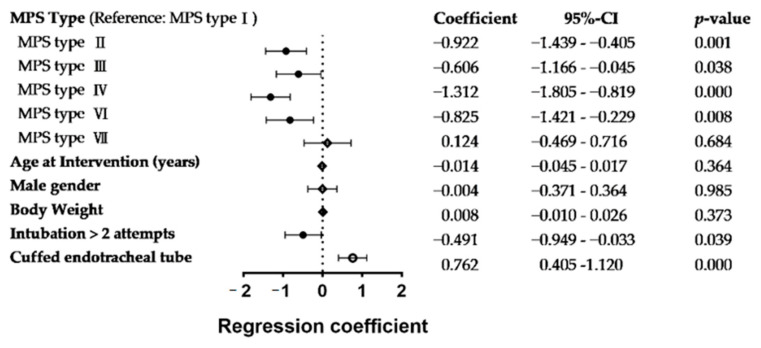
Multivariable linear regression analysis of the independent factors resulting in the differences of endotracheal tube size between the predicted and actual size: a forest plot illustrates the extent of discrepancy on a linear scale; the diamonds/dots and lines indicate the regression coefficient with the 95% confidence interval (95% CI); solid round dots indicate statistically significant with negative effect, open diamonds indicate insignificant findings, open round dot indicate statistically significant with positive effect.

In view of estimating endotracheal tube size for MPS patients, multivariable linear regression was performed to evaluate factors contributing to the differences of endotracheal tube size between the predicted and actual size (Figure 3). Smaller than estimated ETT size was used in MPS type II, III, IV, and VI patients and also in patients who received intubation of multiple attempts. However, a larger than estimated size of ETT was adopted when choosing cuffed ET tubes. Specifically, an average ET tube size is 1.31 mm smaller than that estimated for MPS type II patients and 0.76 mm larger for each cuffed ET tube. Besides, we found no significant relationship between the discordant size of the ET tube and the demographic data of age, gender, and body weight.

### 3.3. Neuraxial Anesthesia

#### 3.3.1. Predominance of Adolescents and Adults

Most patients were male (Table 3), except for one MPS type IIIB female patient that received a gynecological minor surgery. Ages of the most patients were above 12 years old (12/15: 80%), and three patients younger than 12 years old were all MPS type II.

#### 3.3.2. Cardiovascular Events: Two Cases of Hypotension

Hypotensive adverse events occurred following administration of intrathecal 0.5% bupivacaine in two adult patients. One was a MPS type II patient who had aortic stenosis and hypertrophic cardiomyopathy and received 50 μg fentanyl intravenously prior to 7.5 mg intrathecal bupivacaine. He was 110 cm tall and weighed 31.6 kg. The sensory block level was up to T3 with normal oxygenation and respiratory drive, but his blood pressure dropped 25% from the baseline (SBP/DBP: 120/70 to 90/58 mmHg), which was then reversed by three doses of 6 mg ephedrine. Another hypotensive event happened on a MPS type IIIC patient with a height of 160 cm and weight of 47 kg. Normal cardiac function, impaired cognitive function, and musculoskeletal deformity including scoliosis were noted. Induction by mask inhalation of sevoflurane 3–4% was used for accessing the intravenous (IV) route. Then, bupivacaine of 10 mg was given on the lateral decubitus position after hydration with 250 mL normal saline. Escalating systolic blood pressure to the bottom of 50 mmHg without bradycardia or ECG change was noted and then rescued by more aggressive hydration and a total dose of 28 mg ephedrine. After restoring the blood pressure, a low dose of propofol (80–150 mg per hour) infusion after IV administration of 2 mg midazolam and 25 μg fentanyl was given due to uncontrolled movement of his upper limbs. No further hypotension was detected.

#### 3.3.3. Necessity of Premedication and Perioperative Sedation

Before spinal or epidural administration of local anesthetics with possible opioids, nearly half of the patients (7/15: 47%) received premedication or sedation by IV fentanyl, midazolam, ketamine, or sevoflurane inhalation. In addition, seventy-three percent of patients received intraoperative sedation by midazolam, ketamine, propofol, dexmedetomidine, or sevoflurane inhalation. No respiratory compromised event was noted as a result of premedication or sedation. Moreover, levels of sensory bock were not stated in five anesthetic records due to cognitive impairment or perioperative sedation. Local anesthetics were infiltrated on the surgical site for both rescuing prolonged operation and inadequate sensory block.

## 4. Discussion

Our study found the rates of difficult mask ventilation (1.2%; 1/80) and difficult intubation (12.5%) in MPS patients were much less than those in previous studies, since the incidence of difficult mask ventilation was reported as 7–14% and difficult intubation was 28–44% in the systematic review study [4]. Additionally, Dohrmann et al. [19] found the rate of airway management difficulties was 47.8% with direct laryngoscopy, 14.4% with SGA, and 10.1% with indirect intubation. Moreover, 16.7 percent of anesthetic procedures failed the primary airway technique [20], even the conversion from fiberoptic intubation to a different technique was 17.2 percent [19]. In this study, only one episode of conversion from SGA to direct ETT intubation was noted. Considering the high percentage (76%) of upper airway obstruction in MPS patients [5], a thorough preoperative evaluation for the airway is essential to ensure a safer general anesthesia. For each of the MPS patient in our institute, a preprocedural consultation with the experienced otolaryngological doctor is not only routine but a strict rule. Then, adequate preoxygenation, sufficient spontaneous breathing, and maintenance of protective laryngeal reflexes prior to securing the airway [21] would lower the risk of hypoxia. Specifically, a standby ENT team is available for most of the anesthetic inductions. We believe a multidisciplinary experienced team would contribute to the vast quality assurance for safe perioperative airway management.

Based on our knowledge, the investigation of the relationship between the actual size and the estimated size of ETTs for a better prediction is the first report for MPS patients. A previous study revealed that the used sizes of the ETTs were 0.5 mm or larger than those estimated by Cole’s formula in 29% of pediatric patients with congenital heart disease [22]. Our initial assumption was that the chosen size could be smaller than the estimated one in MPS type I, II, IV, and VI due to a more frequent occurrence of progressive abnormal laryngeal anatomy, tracheal deformity, and subglottic narrowing [5]. We found the most significant discrepancy occurred in type IVA patients. However, smaller than estimated size was unexpectedly noted in MPS type III rather than typeI patients, noting that the unlikely difficult airway of type III patients was known both in our and past studies [4,23]. Frequent respiratory tract infection, epilepsy, and difficult swallowing caused by prominent progressive neurological deterioration may result in a narrower airway rather than facial dysmorphism or respiratory tract distortion. Therefore, discordant ETT size and difficult intubation should be separate concerns and implemented in respective strategies. Currently some advanced techniques such as ultrasound [24,25] and printed three-dimensional airway model [26] have been applied for measuring the diameter of pediatric trachea and predicting ETT size. Further investigation of these new techniques applied to MPS patients is necessary.

For more details concerning the specification of ETTs, there has been considerable debate about choosing cuffed or uncuffed tubes, especially for pediatric patients. Based on the presumption of higher complications such as postoperative stridor or tracheal stenosis occurring in cuffed ETTs, uncuffed tubes are preferred for younger children but this is still not conclusive [27]. Our study demonstrated a similar rate of choosing ETTs with or without cuffs for MPS. Interestingly, larger ETTs were adopted while choosing the cuffed ones, which might efficiently decrease the airway resistance because of reduced functional capacity and low lung volumes in MPS individuals [28]. Furthermore, we found the average procedural duration in the cuffed and uncuffed group was 194 min and 154 min, respectively. In brief, lengthy surgeries and optimizing the airway pressure were great concerns in choosing larger cuffed ETTs.

Although there were only 15 neuraxial anesthesia procedures in our 20-year experience, accessible spinal or epidural approaches with a high successful rate for MPS patients has been demonstrated. Musculoskeletal manifestations including scoliosis, dysostosis multiplex, and atlantoaxial instability lead to great difficulties in maintaining patients in a sitting or decubitus position for anesthesia induction. Moreover, poor compliance and involuntary movement of patients caused by intellectual disability and behavior disturbance would deteriorate the situation. Therefore, premedication with anxiolytics and opioids combined with perioperative sedation is a safe strategy for maintaining spontaneous breathing, stable hemodynamics, and a comfortable surgical condition for both patients and medical staffs.

## 5. Conclusions

We concluded that the individualized strategy according to the specific interventions and the different types of MPS will optimize anesthetic safety and perioperative care. Additionally, the specification of endotracheal tube, including size and inflatable cuff, should be implemented in the plan of airway management.

## Figures and Tables

**Table 1:** Summary of anesthesia methods and intervention procedures.

Anesthesia (No./%)	Duration of Anesthesia ^1^ (min)	Type of Procedures	No. of Procedures Total N = 163
**General anesthesia (136/100%)**			148
Bag-Mask (15/11.0%)	137.7 (60–210)	MRI ^2^	12
		ENT ^3^	2
		Orthopedic surgery	1
Supraglottic device (15/11.7%)	103.7 (55–325)	ENT	7
		General or visceral surgery	7
		Orthopedic surgery	1
		Neurosurgery	1
Endotracheal intubation (80/58.8%)	176.2 (60–540)	ENT	55
		General or visceral surgery	19
		Orthopedic surgery	7
		Dental surgery	4
		Neurosurgery	3
		Cardiac surgery	3
Pre-existing airway (26/19.1%)	193.5 (80–440)	ENT	18
		General or visceral surgery	6
		Orthopedic surgery	1
		Neurosurgery	1
		MRI	1
**Neuraxial anesthesia** **(15/100%)**	105.7 (45–190)	General or visceral surgery	14
		Orthopedic surgery	1

^1^ Data are expressed as mean (range). ^2^ MRI: magnetic resonance imaging. ^3^ ENT: ear, nose, and throat.

**Table 2:** General anesthesia and patients’ characteristics.

Classification	Total Patient	Type I	Type II	Type IIIA	Type IIIB	Type IIIC	Type IVA	Type VI	Type VII
**Episodes of anesthesia**	136	26	33	2	15	2	35	12	11
**Gender (M/F)**	97/39	9/17	33/0	2/0	13/2	2/0	29/6	9/3	0/11
**Age (y/o) ^1^**	13.7 (0–38)	15.2 (0–35)	12.8 (2–25)	9	5.7 (2–14)	26	20.2 (6–33)	12.6 (6–26)	3.4 (1–6)
**Body weight (kg)**	27.4 (4.4–84)	38.4 (4.4–84)	29.1 (15–53)	25	21.6 (15.7–36)	42	26.8 (13–39.3)	19.9 (13–33)	11.6 (8–16.3)
**Body height (cm) ^2^**	112.1 (58–167;94)	128.1 (58–167;21)	125.9 (98–158;17)	n.a. ^4^	108.1 (99–140;11)	160	99.6 (84–129;30)	94.6 (86–110;7)	91.4 (86–99;6)
**Body mass index (kg/m^2^)**	23.4 (11.8–49.4)	22.2 (11.8–31.6)	21.1 (12.8–27.9)	n.a.	18.1 (14.4–20.0)	16.4	29.7 (13.2–49.4)	20.7 (17.6–27.3)	17.4 (16.6–19.1)
**Bag-Mask**	15	1	2	0	9	0	1	1	1
**Supraglottic device**	15	2	6	0	1	0	2	2	2
**Pre-existing airway**	26	2	10	0	1	0	12	1	0
**Endotracheal intubation**	80	21	15	2	4	2	20	8	8
***Direct laryngoscopy***	34	9	3	0	3	0	8	3	8
***Video-assisted***	18	3	4	0	1	2	8	0	0
***Fiberoptic or bronchoscope***	28	9	8	2	0	0	4	5	0
**(C/L) III° or IV° documented ^3^**	28	16	5	0	0	0	4	3	0
**Difficult airway management**	12	4	3	0	0	0	2	3	0
**Respiratory adverse event**	3	0	1	0	0	0	1	0	1
**Cardiovascular adverse event**	5	1	2	0	1	0	1	0	0
**Postoperative ICU ^5^ care**	54	10	17	1	5	0	13	4	4

^1^ Data are expressed as mean (range). ^2^ Data are expressed as mean (range; available data No.) due to unavailable measurements of body height in some patients’ records. ^3^ (C/L): Cormack–Lehane classification; It was documented in 60 anesthetic records. ^4^ n.a.: not applicable ^5^ ICU: intensive care unit.

**Table 3:** Neuraxial anesthesia: general data, details of spinal anesthesia, and hypotensive event.

Classification	Type I	Type II	Type III	Type IV	Total Patient
**Subtype: number**	I(S):1	11	IIIB:1 IIIC:1	IV(A):1	15
**Fender (M/F)**	1/0	11/0	1/1	1/0	14/1
**Age (y/o) ^1^**	25	15.8 (7–23)	21 (17–25)	28	17.9
**Body weight (kg)**	74	37.8 (24–57)	43 (39–47)	26.6	40.2
**Body height (cm)**	161	133.5 (110.5–156) ^2^	152 (144–160)	h104	136.4
**Dose of intrathecal 5% bupivacaine (mg)**	13	8.4 (5–11)	10	7.5	8.9
**Combine with epidural anesthesia**	0	1	0	0	1
**Premedication for analgesia or sedation**	0	6	1	0	7
**Intraoperative opioids**	0	5	1	1	7
**Intraoperative midazolam or ketamine**	0	7	1	1	9
**Intraoperative sevoflurane or propofol**	0	2	1	0	3
**Intraoperative local anesthesia**	0	3	0	1	4
**Duration of surgery (min)**	170	100.5 (55–190)	77.5 (45–110)	155	105.7
**Hypotensive adverse event**	0	1	1	0	2

^1^ Data were expressed as mean (range) for multiple values. ^2^ Two data were not available from all medical records.

## Data Availability

All data are present within the article. The individual data registered in our electronic anesthetic records and quality assurance/improvement (QA) system from the Anesthesiology Department of Mackay Memorial Hospital will be available upon request.
